# Alignment Solution for CT Image Reconstruction using Fixed Point and Virtual Rotation Axis

**DOI:** 10.1038/srep41218

**Published:** 2017-01-25

**Authors:** Kyungtaek Jun, Seokhwan Yoon

**Affiliations:** 1 Seoul Hayannara Research Center, Gyeongsangnam-do, Changwon-si, 51495 Korea; 2 Department of Dentistry, Seoul National University, Seoul, 03080 Korea

## Abstract

Since X-ray tomography is now widely adopted in many different areas, it becomes more crucial to find a robust routine of handling tomographic data to get better quality of reconstructions. Though there are several existing techniques, it seems helpful to have a more automated method to remove the possible errors that hinder clearer image reconstruction. Here, we proposed an alternative method and new algorithm using the sinogram and the fixed point. An advanced physical concept of Center of Attenuation (CA) was also introduced to figure out how this fixed point is applied to the reconstruction of image having errors we categorized in this article. Our technique showed a promising performance in restoring images having translation and vertical tilt errors.

X-ray tomography is considered more than essential nowadays in various fields^1^, and combined with advanced X-ray sources, it provides more sophisticated scientific insights^2^. As Wang *et al*.^3^ once argued, it is desirable to achieve an automated data processing with minimum human interaction due to the demand of high-throughput experiments. Given the fact that we only get the 2D projections from the x-ray penetration to form the internal 3D structure of a sample, a number of scientists have shared their insights to get rid of impairing effects in getting a better quality of reconstruction, translation and tilt errors, for example.

They have focused on the determination of the center of rotation (CoR), and there are mainly three families of methods. The first category uses pairs of projection images taken from the reverse viewing angles (at 0–180 degrees)^4^. The second method evaluates the projection image from the image reconstruction by using a parameter to measure the quality of image reconstruction and to calibrate the relative offset of the rotation axis^5^. The last one considers the center-of-mass (CM)^6^. In the previous researches^7^,^8^, researchers tried to find the rotation axis (RA) by projection image rearrangement, but there was no proper method to find the RA even in 2-dimensional space^9^. Hence it is always useful to figure out a new method and algorithm to correct the flawed information to obtain a reconstruction of better quality.

In this article we propose an alternative approach to remove the errors, specifically translation and tilt errors by using the sinogram and the fixed point. Wherein, fixed point is defined as the point in space that can be discriminated or calculated by analysis of a projection image set. The actual RA is located on the center line of the sinusoidal trajectory of the Fixed point (FP). Furthermore, we did not try to determine the CoR; instead, we aligned the vertical center line of projection to the CoR to make it like a virtual RA, which was enabled by our algorithm.

## The relative position of an object in the real space and the sinograms in 2D

A sinogram involves the information about a specific layer of an object and accumulates the projection shadows taken from each projection angle to build a reconstruction of the layer. This sinogram will later be transformed into a reconstruction through the inverse Radon transform. Apparently, an errorless reconstruction will be obtained only with an ideal sinogram or an ideal projection set, and a necessary condition for the ideal sinogram is that its pattern is to be changed sequentially depending on the projection angles.

**Assertion 1** *When the location of an object is changed on the stage, the sinogram pattern will be also changed while the projection shadow from an angle remains the same.*

There might be slight differences in the projection shadow due to the limit of image digitalizing techniques; this can be approximated by a linear interpolation. Where a projection shadow lies on the sinogram is closely related to where an object lies in the real space; this relationship should be first investigated before we further use sinograms for our analysis. The object on the rotating stage is known as to rotate around the rotation axis (RA) and it means that any single point of the object has its own circular trajectory, which will be expressed as a sinusoidal function in the sinogram. The point on the RA will be the straight line across the center of the sinogram in ideal condition. Our hypothesis is that if we have the projection set of a specific object and if we know the ideal sinogram pattern of this object, we can modify the actual sinogram pattern to meet the ideal one and consequently get the errorless reconstruction. By modifying the sinogram, we can make the same effect just like moving the specimen in real space from where it was to the center on the stage, while the recorded image is preserved. This saves the current efforts of determining the CoR and setting the specimen on the RA, and the simple modification of sinogram will yield the same result. (see Supplementary Section 1).

### 2D Correction Algorithm: Virtual Focusing Method using FP

In the actual settings of computed tomography (CT) system, the translation error is not only either a horizontal or vertical error but also a combination of both. Thus, an optimal solution for the orthogonal translation error correction (see Supplementary Section 2.2) is not enough in general. The horizontal shift of an object is hard to be detected on the projection image and so, difficult to be calculated. This leads us to a need of approaching this issue by adopting a fairly new concept and a point of view. Knowing that the errors in the actual settings are complicated and often a mixture of different types of errors, one should start with understanding and defining the relationship between the real space and the sinogram, which is actually a projected and reconstructed version of real space, so as to use it to draw a general and automated method of correction in advance.

Ideally, the center line of a sinogram is made up of the projection of RA. Any single point in the real space will move on a circular trajectory rotating around the RA and this will correspond to a function on the sinogram. Let’s say there is a point *p* and it moves on a circular trajectory, then this point *p* will be transformed into a curve on the sinogram and represented by the sinusoidal function *T*_*r,φ*_.

**Assertion 2** *When an object rotates around RA, the circular trajectory for a point p of the object can be represented by a curve in the sinogram.*


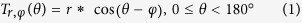


**Figure 1:**
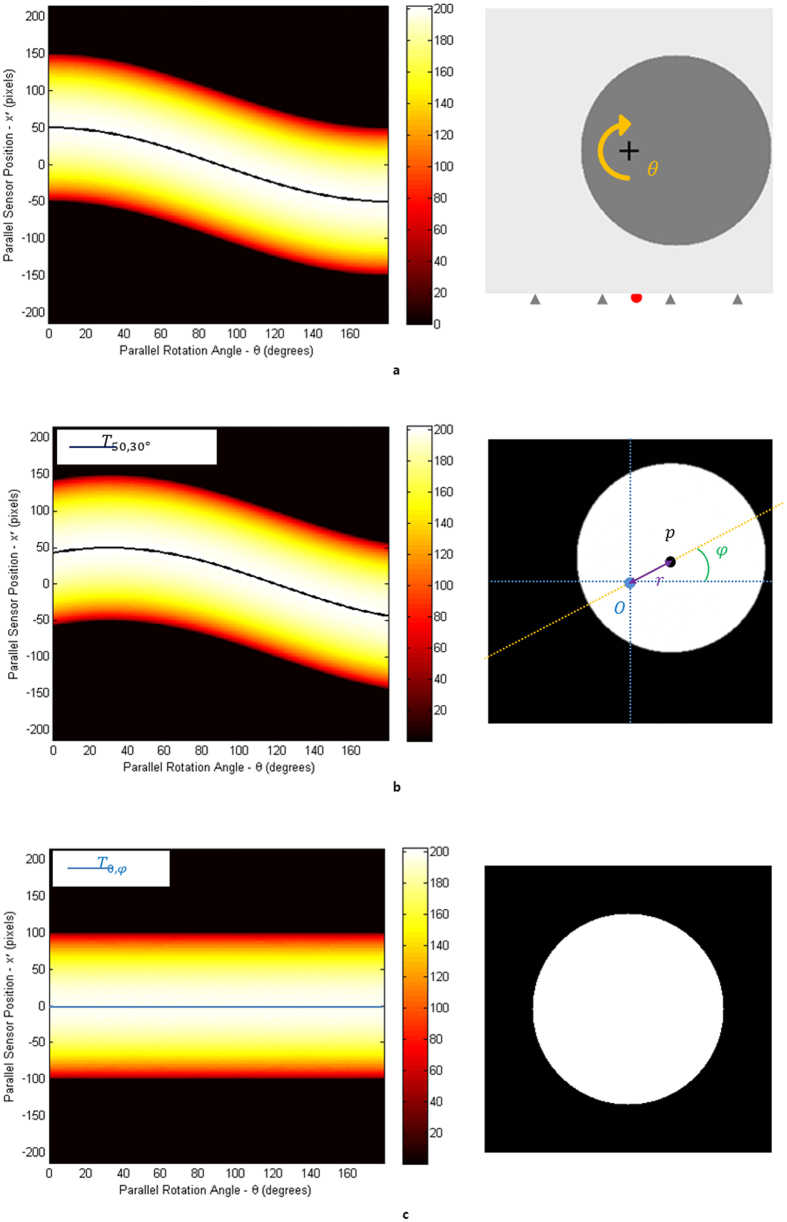
The sinograms of circular image specimen and its reconstructions. Notice that we marked the stage with the red dot at the bottom to indicate ***θ*** is zero degree. (**a**) The sinogram when the specimen (right panel) is translated in parallel with the beam from the center of the stage at ***θ*** = 0°. (**b**) The sinogram and its reconstruction with which we figured 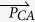 of each column in sinogram a and align them on the function *T*_50,30°_. 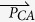 was marked in black on each column of the sinogram. Because the 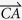 was off the center of the stage, the black line showed sinusoidal function. The reconstruction is moved to the upward side of the original stage. (**c**) The sinogram and its reconstruction with which we figured 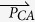 of each column in sinogram a and align them on the function *T*_0,*φ*_. 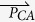 was marked in blue on each column of the sinogram. Because 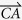 was moved to the center of stage this time, it appeared as a straight line across the center. The center of reconstruction of specimen is moved to the center of the original stage. This modification shows the same result just like moving the specimen in real space.

We defined *r* as the distance between the rotation axis and the point *p. θ* is the projecting angle, and *φ* is the angle between the line 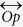 and the orthogonal line to the projection angle at *θ* = 0 (Fig. 1b). In ideal cases, the center *O* is converted to *T*_*0,φ*_ in the sinogram, but not in the actual sinogram.

Using Assertion 1 and 2 which were mentioned earlier in our study, we can alter an inaccurate sinogram to a correct one. *T*_*r,φ*_ is a function that shows how a specific point *p* in the real space moves on the sinogram. It means that we can keep tracing a point of the real space on the sinogram and use it further as we intend to. In other words, if a point *p* in the solid specimen rotates on the stage and the projected curve drawn by the movement of *p* for each angle is the same as the sinusoidal curve by *T*_*r,φ*_ in the sinogram, then the projected trajectories of other points in the specimen should satisfy the projected curves by 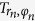 and all points in space satisfy the Helgason–Ludwig consistency condition. Being the center of a circle, the point *p* on the sinogram in Fig. 1a will be always on the center of the projection shadows. It follows the curve marked as a black line on the sinogram since the specimen itself is off the center. The point *p* is a center-of-mass for a circular object if the object has an identical medium and acts as a fixed point to represent the same spot even when the projection angle changes. With the point *p* translated on the function *T*_*0,φ*_, that is to say, with each column in the sinogram moved so that the point *p* is on the center and the black lined curve is laid linearly on the center line of the sinogram (Fig. 1c), the center of an object will match exactly to the center of projection and the sinogram will become linear. When applied to a real space, this virtual translation has the same effect as we physically set the center of an object on the RA.

Nevertheless, actual objects are not always cylindrical. They rather come in much more complicated structures, and as a result, defining a fixed point on the projections is not an easy issue. We came up with an advanced physical concept called the center of attenuation (CA) to make this issue simpler. The CA is a similar idea with the center-of-mass, but is different in a way that a unit particle of an object is expressed not by the X-ray density but by the x-ray mass attenuation coefficient^10^ (MAC) which is measured by the unit voxel. In general, the center of mass estimated by X-ray densities in projection images can’t play a role in our FP because of the followings: the X-ray absorption for the thickness of specimen is not linear and the specimen size is larger than the charged coupled device (CCD). Our assumption for using MAC is that there is co-relationship between a unit voxel of actual object and a unit voxel of reconstruction (see Supplementary Fig. S7). For 3D, the CA calculation requires the object part in a subset of common layers. (We will discuss the common layer in Method).

**Assertion 3** *When the relationship between the absorption of photons and the thickness of specimen is not linear after the x-ray beam penetrates the specimen, it should be first transformed into something with linearity and then utilized to figure out the CA.*

**Figure 2:**
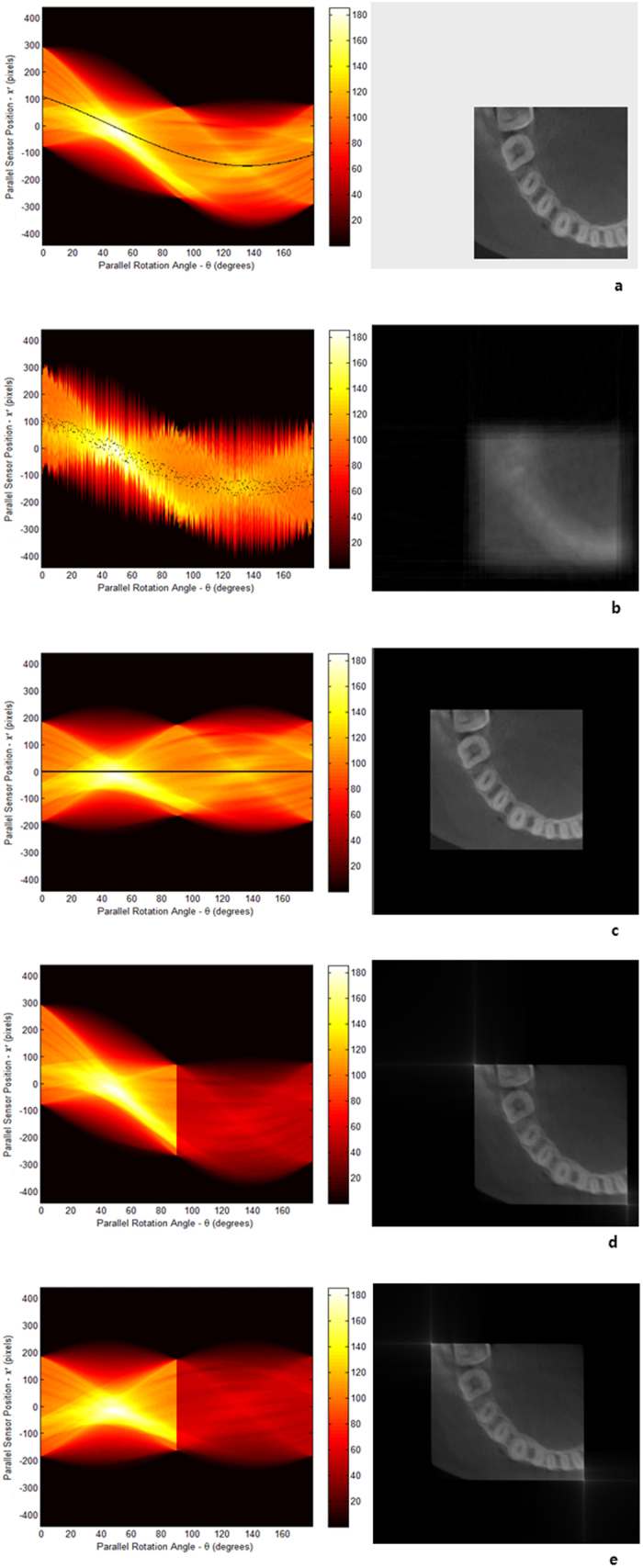
Analysis of virtual focusing method of a partial image of human lower jaw including the teeth. (**a**) An image sample (right panel) and its sinogram (left panel) (The object is on the lower right side of the stage). The 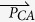
s of all projections are marked in black in the sinogram. They follow the circular trajectory in real space, therefore show the sinusoidal graph in the sinogram. (**b**) A sinogram (left panel) with artificial translation errors added to the sinogram (**a**) including vertical and horizontal movement at each projection angle and its reconstruction (right panel). The 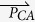
s, the black marks, are all scattered. (**c**) A sinogram that we aligned 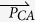 of the sinogram (**b**) on *T*_0,*φ*_ and its reconstruction. The 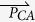
s are arranged linearly on the center of the sinogram. 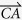 is on the center of the stage and the image is ideally restored. (**d**) The cases when the x-ray density of projection is changed during the beam time. The sinogram and its reconstruction when the x-ray density of projection in **a** is decreased by half after *θ* = 90°. The pattern of ideal sinogram of **a** was maintained even though there was 50% decrease in x-ray density from the 90 degrees of *θ* while preserving the linear relationship. (**e**) The sinogram and its reconstruction when the CA was applied to (**d**). The reconstructed using the sinogram with the 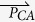 and the T_r,φ_ function applied showed no difference in terms of image itself when compared with the reconstruction of (**d**), and the image of specimen was laid in the center.

When the value of MAC is changed into the one expressed in length to calculate the CA (See Method). For the calculation of the CA, all pixel values obtained by X-ray density in projection should be expressed to the sum of MAC. For the calculation of the CA, all pixel values obtained by X-ray density in projection should be expressed to the sum of MAC. When an x-ray beam is shot to an object, the object absorbs certain amount of the energy and the rest of attenuated energy arrives on the detector. Then we get the projection image. We assumed that there exists an invariable center of MAC for a certain object, just like every object has a center of mass obtained by classical mechanics, and it is fixed on a certain spot, either inside or outside of the specimen, acting as a fixed point that does not change depending on the projection angle. This fixed point can be calculated from the projection images obtained from each angle of x-ray penetration. It will lie on the sinogram satisfying the *T*_*r,φ*_ function that was mentioned earlier and this is how we get an ideal sinogram pattern. Figure 2 shows how we applied this idea of CA to the image reconstruction.

### Analysis of 3D Image Reconstruction from a Projection Image Set

There are only translation errors in the two dimensional projections. However, other types of error have to be taken into consideration when it comes to three dimensions. Mainly, three types of errors can be discussed; a translation error caused by the shift of a specimen, a tilt error caused when the RA is tilted, and the rotation error that is caused when a specimen spins on its own axis.

A translation error is the error that occurs when the object is moved by any chance during the beam time, and this movement can be in three directions in the 3D space. A compensation for this error can be done through just the way we did with the translation error in 2D space, bringing projected fixed points on the ***T***_r,φ,h_ function (*h* is a height of a specific layer of projections. See Method).

In 3-dimensional space, it is important to consider tilt errors because a tilted image carries information from different layers and consequently induces a flawed image reconstruction. Thus, if we compensate the tilt error, it means that we make one layer to carry all the information about a single part of an object. To categorize and analyze tilt errors, we need to think about the object itself and the stage that the specimen is placed on. We will not call the case of tilted object an error. It is because we can make a reconstruction without any correction procedures in this case (see Supplementary Section 3.1). When it is the RA that is tilted, one should make sure that the tilt error is corrected.

**Figure 3:**
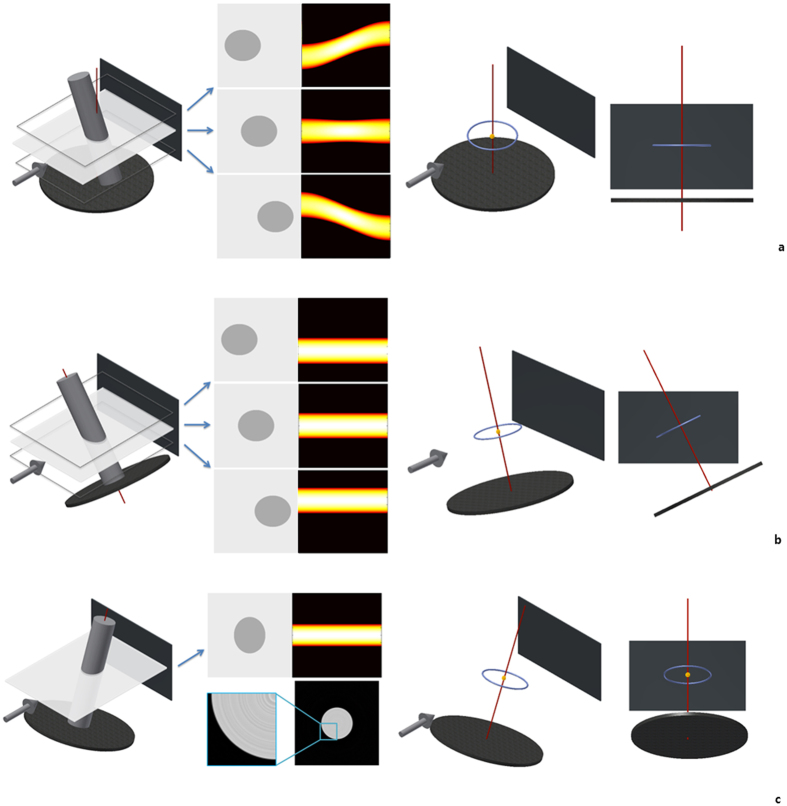
The sinogram pattern of cylindrical specimen and the 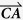 trajectory, depending on the tilt of the object or the RA. The third and fourth pictures in (**a**,**b** and **c**) show the trajectories of 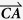. When the 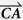 is on the RA (orange dot in the picture), it is expressed as a dot. In other cases, it is expressed as a circular trajectory (blue circle). (**a**) The sinogram and the 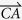 trajectory when an object is tilted. Even though the object is tilted, the 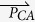 is on the parallel line with the stage in the projection. This is quite a typical occasion where no error is found. (**b**) The sinogram and the 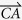 trajectory when the RA is vertically tilted. The object rotates around the RA and the line that 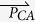 makes are perpendicular to the RA on the projection. (**c**) The sinogram, its reconstruction and the 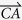 trajectory when the RA has a parallel tilt. The pattern of the sinogram is linear around the center. However, its reconstruction is flawed (shown better in the magnified figure), since the layers are all mixed up at each angle θ. In this case, the collection of 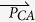
s in the projection makes an elliptical shape, not a line. The RA is also perpendicular to the major axis of 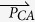 trajectory in this case.

In terms of the tilted RA, we can classify this into two cases of tilt errors in detail; one is of a vertical direction and the other is of a horizontal direction to the x-ray beam. The former is shown in the Fig. 3(b) and the latter is shown in the Fig. 3(c). In a vertical tilt, the polar angle increases when the azimuthal angle is either 90 or 270 degrees. On the other hands, the polar angle increases when an azimuthal angle is either 0 or 180 degrees in a parallel tilt. The azimuthal angle increases counter clockwise against the beam. The ideal RA which stands upright without any tilt error is assumed to be the reference for the polar angle.

It is the case of a vertical tilt when the RA is tilted against the axis of whole projection and the stage is rotating with the tilted RA. If we assume that the common layer is also tilted in parallel with the stage, namely tilted by the same angle in the projection, the projection information is now about a single layer, not mixed with the ones about other layers. Hence, if we only know how much the RA is tilted, and carry out a rotation compensation on the whole projection rotating around the CA, we can find the common layer that we want to concentrate on (see Method). Contrarily, the parallel tilt carries information about more than one layer and it is hard to get an ideal sinogram pattern in this case.

## Discussion and Conclusion

In this study, we categorized the errors that possibly take place during the beam time. Although there certainly is a limit to what extent we can correct the reconstruction in each case, we can modify most of the errors using FP and enable the improvement of the reconstruction quality. We call the reconstruction as the ideally focused reconstruction from now, which is coming from ideal sinogram pattern. There are two cases for the tilt errors. When it is the vertical tilt, we get the ideally focused reconstruction. Meanwhile, we will not be able to get an ideally focused reconstruction with the projection set for the parallel tilt since the information of different layers are mixed up. The tilt error is mostly a mixture of both vertical and parallel tilts; the optimized projection set in which the vertical error is compensated is the best deal in this case. For the rotation error, it seems impossible to reconstruct images appropriately because we hardly get the complete projection set.

**Figure 4:**
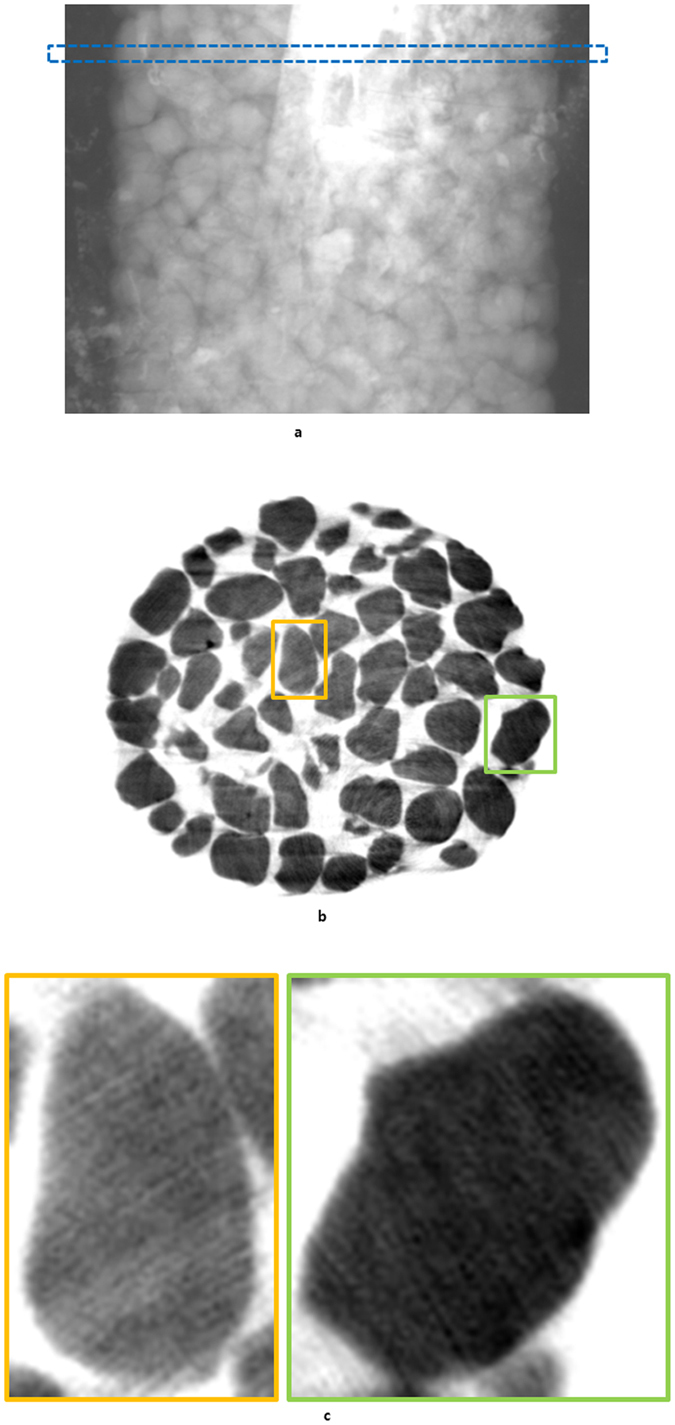
A reconstruction influenced by high density areas, and a projection image which cannot preserve the linear relationship between the absorption of photons and thickness of the specimen. (**a**) The white parts in the projection image represent the high density areas. The marked rectangle represents a common layer including high density areas. (**b**) This reconstruction was made by ideal sinogram pattern, but the density of the grain depends on the position of the reconstructed object. (**c**) Two magnified images in the reconstruction of (**b**).

The CA we suggested in this study will function as the fixed point that is one of the intrinsic factors of an object. It works as an invariable point inside (sometimes outside) of an object which does not change even when the object is translated, or the RA is tilted. Nonetheless, we have to calculate this CA from the projected image so as to utilize it, which results in one limitation that the intensity variation of the beam reaching each cell of the CCD in the formula for 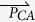 should be linearly proportional to the length of the specimen. That is, the value *α*_*k*_ of each cell which consists of the image from the projection should have certain linear relationship with the length of the specimen, or at least should be changeable to have linear relationship (see Supplementary Section 5 and Fig. 4). Particularly in soft X-ray tomography (SXT), there is a linear relationship^11^ and a unique linear absorption coefficient measurement is given^12^,^13^. Scientists have successfully used imaging of SXT for 3D images of various cell types with isotropic resolution^12^,^14^,^15^,^16^,^17^,^18^,^19^. They have tried to automate the tracking process of fiducial markers through a stack of projection images^20^,^21^,^22^,^23^,^24^,^25^. The trajectories of these fiducial markers follow ***T***_*r*,φ,h_ function; the fiducial markers can also be used as fixed points.

To obtain a well reconstruction, we need to get a stable x-ray density against the object regardless of projecting angles. In reality though, we sometimes get the different x-ray density according to the projecting angle changes, and then, the value of *A*(see Method) also should be changed for the formula applying 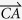. It would be difficult to generally use 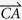 for the image reconstruction if the location of CA depends on the changes of x-ray density and the *A* value. The changes of x-ray density (according to the angle) will appear in forms of the shadow intensity in the sinogram. Nonetheless, it won’t affect the pattern of the sinogram. Figure 2d and e proves to us that the location of CA does not vary despite the changes of *A* value against each *θ* caused by the different x-ray density. We may be able to get a better image if we mathematically modify the x-ray density in order to even up the *A* value. Furthermore, if we can find the minimum value of *A* preserving the linearity, we can obtain sufficiently large value of *A* by mathematical modification. For the total amount of radiation dose, we can get the better reconstructions by increasing the total number of projection images with low dose X-ray. When the specimen is longer than the CCD, its whole image is not projected and consequently, there would be a limitation in applying the CA. We expect to discover another solution even in those cases if we can figure out the common layer on which the object is commonly projected and apply the CA on it.

**Figure 5:**
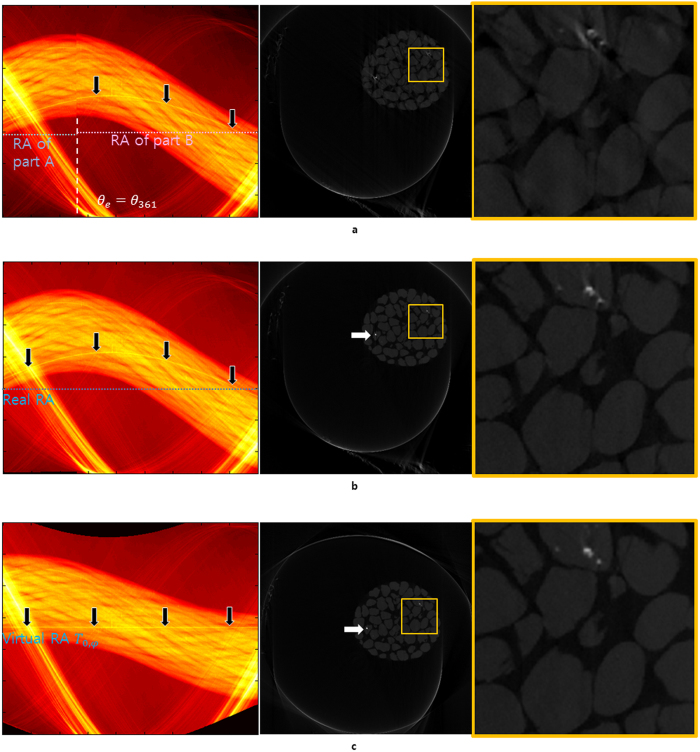
The sinograms and reconstructions of a layer of Hanford soil in a polyether ether ketone(PEEK) column from National Synchrotron Light Source (NSLS) X2B beamline at Brookhaven National Laboratory (BNL). The third pictures in (**a**,**b**, and **c**) are the magnified images of the yellow frames of the corresponding reconstruction s in the middle. (**a**) From the analysis of this sinogram, the specific *f*_*n*_(*t*) value was found at the discontinued spot; it was expected that a vertical translation error arose between the 360th and the 361th projections. For the middle panel, we used the RA of part B to minimize the error from two distinct RAs. (**b**) We corrected a vertical translation error (see Supplementary Section 2.2) to the part A of the sinogram (**a**), which brought us a sinogram with the continuity and a better reconstruction. The area with high x-ray impermeability is moving along inside the sinogram showing a sinusoidal pattern (black arrows in left panel). In its reconstruction, this area is placed in the upper right part (white arrow) in the middle panel. (**c**) The center of the x-ray impermeable material is set as the fixed point and it is applied to the function *T*_0,*φ*_ representing the virtual rotational axis of the sinogram; the FP is now shifted on the line across the center in the sinogram (black arrows). It is placed on the center of the image in the reconstruction image (white arrow).

In spite of the benefits of categorization, the errors often come about not as we categorized, but as a mixture of different types of errors. It is not possible to perfectly restore the image even with the CA and the ***T***_*r*,φ,h_ function in those cases. The application will be much more extended if we can discover more fixed points besides CA and we can utilize each of them to the ***T***_*r*,φ,h_ function. Although further researches should be proceeded, we simply showed the example of this idea in Fig. 5. If there is an area whose boundary is definite and x-ray impermeability is comparatively high, it will be distinguished in most of projections. We might be able to use its center as another fixed point. We searched for the trajectory of the fixed point in the sinogram of Fig. 5(a), and corrected a vertical translation error as Fig. 5(b) (See supplementary Section 2.2). The fixed point was transformed into the function ***T***_0,φ,h_ in Fig. 5(c). Here, we see that it provided us with a clearer image when the ***T***_0,φ,h_ function was applied to the fixed point. It is expected most of the errors will be corrected when the fixed point and the CA applied to the ***T***_*r*,φ,h_ function in case of mixed errors.

Additional researches are needed because of the limits of applying the CA to real images. We will continue to seek for the correct algorithm in applying the CA and find out more traits that can be used as the fixed points. This endeavor will contribute to getting better reconstructions even when the various errors coincide, when the whole image is not obtained because the specimen outlies the CCD, and when the parallel tilt error is included.

## Method

The calculation of CA is very similar with the one for center-of-mass obtained by classical mechanics. First, a virtual rectangular coordinate and unit cubes are adopted in real space and each vertex is the whole number on the coordinate. Let’s assume that the specimen placed in the coordinate is composed of *n* unit cubes. The *k*-th cube among the *n* cubes of the object has a mass attenuation coefficient *μ/ρ* (MAC) for the given unit cube related to voxel length on its designated location and it is represented as *a*_*i*_, which can be linearly calculated through a series of calculation processes. Other coefficients related to X-ray transmittance which might represent the specificity of material, such as mass energy-absorption coefficients, can be used for the same purpose.

Below is the formulation we used to mathematically identify the location of CA on the coordinate.


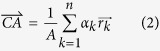


*α*_*k*_ is 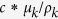, and c satisfy that an unit voxel density for the given initial energy has the same value of *α*_*k*_. *A* is the total sum of *α*_*k*_ and a constant value. *A* is defined in a subset of a whole common layer set (this will be discussed in 3D) and *n* is the total grid number of the object in a subset of common layers of the real space. 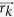 indicates the center of *k*-th grid. 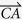 is one of the fixed points in real space and particularly significant in that it will be always projected and we can trace it later on the projections. So, 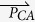, a projected position of 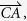 can be calculated in the projection image as below


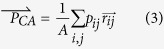


Here we see that 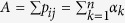, and *p*_*ij*_ is the modified (*i, j*) pixel value of the projected specimen in the 2-dimensional projection and 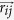 is the center of the pixel. As the *α*_*k*_ is calculated by MAC, the *p*_*ij*_ also should be linearly expressed and modified by the sum of the related *α*_*k*_.

In the projection image, the MAC of the area except for the computational domain for the object shadow should be zero in the ideal status. If it is not zero, 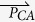 might not be able to act as the fixed point. So we need to modify the attenuation value of this area to have at least the average of zero when it is not exactly zero, ensuring the same amount of the value is added to or subtracted from the *p*_*ij*_ area.


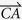
 in real space becomes a specific point that we know and it is projected to be 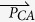 and expressed as the *T*_*r,φ*_ function on the sinogram. The center of projection is always the center line of the sinogram. Therefore, when 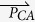 from each angle of x-ray beam is translated onto the center line *T*_0,*φ*_ of the sinogram, it can be considered the same as the fixed point of an object is placed on the RA. In other words, we are able to get rid of all the translation errors in the 2-dimensional space by translating 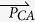 of each projection on the *T*_*r,φ*_ function. Since 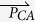 of each projection is one of the projected fixed points, all fixed points can be translated on *T*_*r,φ*_ and expressed in the sinogram. For actual samples, the center line of the sinogram doesn’t represent a RA. In this case, we define that the center line is the virtual rotation axis representing *T*_0,*φ*_, and rearrange any fixed point on *T*_*r,φ*_ to make one of ideal sinograms. Especially, we translated the fixed point on *T*_0,*φ*_ as shown in Fig. 5c.

### 3D translation error correction

A translation error is the error that occurs when there is a certain movement of the object during the beam time. This movement can be directed to three directions in the 3D space. A compensation for this error can be done through similar way we did with the translation error in 2D space, bringing projected fixed points on the ***T***_r,φ,h_ function.


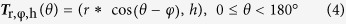


where *h* is a level of a common layer of a projection set. We define a common layer as the plane that envelops the same whole part of the object’s axial level in the real space and perpendicular to the RA.

**Figure 6:**
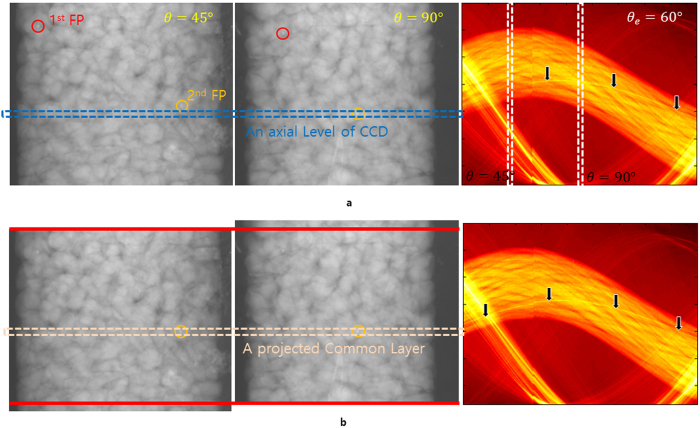
Rearrangement of the projection images via the common layer. (**a**) Projection images at θ = 45° (left) and at *θ* = 90° (middle). Right panel shows the sinogram with errors (*θ*_*e*_ = 60°) at the blue colored axial level of the CCD. These projection images (at *θ* = 45° and *θ* = 90°) contain two cleared fixed points (red and orange circles indicate the first and second fixed points, respectively). (**b**) Adjusted projection images via the second fixed point. The region between upper and lower red colored lines is a common layer set. A sinogram of each common layer can be converted to the ideal sinogram pattern. The right panel shows the sinogram at the projected common layer containing the second fixed point. The black arrows indicate the trajectory of the second fixed point in the sinogram.

The translation error in 3D is different from the one in 2D in terms that one should consider layers from where the error occurs. Our numerical results illustrate how the translation error can be compensated by using common layers which include fixed points (Fig. 6). Figure 6(a) shows the sinogram with translation errors (*θ*_*e*_ = 60°) at the blue colored axial level of the CCD (right panel) and two projection images at *θ* = 45° and *θ* = 90°, (left and middle panel, respectively). In order to fix the error, we first set two cleared fixed points in the projection sets at both angles (red and orange colored circles are the first and second fixed points, respectively). However, those fixed points were placed on different axial level of the CCD. For this case, we adjusted the projection images via the high density areas by rearranging the projection set where each high density area is located on the same axial level, called the common layer (Fig. 6(b)). Once this process is completed, the high density area appears its trajectory in the sinogram over *θ* less than 60°. The shape of the high density area can be considered as a circular shape and the center of the circle can be a projected fixed point. To fix the translation errors, we translate the projected fixed points onto the *T*_*r,φ,h*_ function of the common layer. To make our calculation easier, we place the projected fixed points on the *T*_0,*φ,h*_ and then the projected fixed points are located on the projected RA. Adjusting common layers is a significant procedure in the 3-dimensional image reconstruction because it is simply not possible to reconstruct an image well using projections of different layers.

### Correction algorithm for the vertical tilt of a RA

In correction process, the priority is to know by which angle the RA is tilted. The whole projected images will be corrected accordingly to the tilted angle using the vertical center line of whole projection as a fiducial line. To calculate the specific angle that the RA is tilted by, we used the FP in this study. Assuming that we virtually collected the every projected fixed point of each projection we get from a specimen and placed them on the CCD, we thought that they would make a virtual line segment just like in Fig. 3b. In fact, the line segment of projected fixed point stands for the trajectory of FP, and the tilted RA is perpendicular to it. If this RA and the vertical line make a certain angle, it is the angle by which we will rotate the projection set to meet the ideal one which doesn’t have the tilt error.

### Calculation of tilted angle of RA in parallel tilt errors

Even in a parallel tilt, the tilted angle can be calculated with the same method. The angle of parallel tilt, α, satisfies 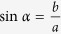. Here, *a* means the major axis and *b* means the minor axis against the trajectory of CA (Fig. 3c). Whether the orientation of trajectory in the projection is clockwise or counter clockwise depends on whether the azimuthal angle of RA is 0 degree or 180 degrees. This method can be also applied to the mixed error of vertical and parallel tilts. (see Supplementary Fig. S6).

*For access to the source code implementing the algorithm, please contact the corresponding author*.

## Additional Information

**How to cite this article:** Jun, K. and Yoon, S. Alignment Solution for CT Image Reconstruction using Fixed Point and Virtual Rotation Axis. *Sci. Rep.* **7**, 41218; doi: 10.1038/srep41218 (2017).

**Publisher's note:** Springer Nature remains neutral with regard to jurisdictional claims in published maps and institutional affiliations.

## Supplementary Material

Supplementary Information
