# Supplementary material for: Alignment Solution for CT Image Reconstruction using Fixed Point and Virtual Rotation Axis
Source: Sci Rep. 2017 Jan 25;7:41218. doi: 10.1038/srep41218 (PMC5264594; doi:10.1038/srep41218)
Supplement: Supplementary Information [file srep41218-s1.pdf]

Alignment Solution for CT Image Reconstruction using Fixed Point and Virtual Rotation Axis

KyungtaekJun[a,*], SeokhwanYoon[b]

[a]SeoulHayannaraResearch Center, Gyeongsangnam-do,Changwon-si 51495 Korea

[b]Department of Dentistry, Seoul National University, Seoul 03080 Korea

*ktfriends@gmail.com

Contents

1. The relationship between the sinogram and the real space

2. Translation error

2.1. Continuity & Discontinuity by an Object movement and a CT system Vibration

2.2. An optimization algorithm of translation error

2.3 The limit of optimization

3. Tilting error

3.1 The distinction between the tilt of RA and the one of the object in tilting errors.

3.2 tilting error

4. Rotation errors

5. The linearity of x-ray absorption depending on the thickness of an object.

1. The relationship between the sinogram and the real space

In this report we propose an alternative approach to correct those errors, mainly translational and tilting errors, using the sinogram and the fixed point. We focused on the fact that there must be a "not-moving" point in the projected image of a specimen just like the concept of center-of-mass. It is hardly possible to determine the CoR of the specimen because, even though the RA hasn't changed, it looks like changed when the specimen itself was rotated. We did not seek to determine the CoR; instead, we aligned the vertical center line of projection to the CoR to make it like a virtual RA, which was enabled by our algorithm.

The sinogram was constructed by a projection image of the circular specimen, a cross-sectional image of a cylinder in 2D. Fig. S1a shows when the specimen was located on the center of the stage. The stage rotated clockwise for the entire 180° or 360°, and the x-ray beam was fixed at $\theta = 0$. The sinogram pattern is linear in this case. One can easily spot the changes of the sinogram pattern when the cylinder was moved toward the parallel beam on the stage in Fig. S1b, and when it was moved to the left from the beam in Fig. S1c. The projection shadow from an angle remains the same, even when the specimen was moved to a different spot and had a different sinogram pattern.

2. Translation error

2.1 Continuity & Discontinuity by an Object movement and a CT system Vibration

Assumed that the projection images are all clear, it is the translation error that we can first think of in the 2D space during the beam time. We categorized translation errors into two; the orthogonal translation and the parallel translation, which are the two elements in the basis that we decided to consider in this study. By the orthogonal translation, we mean that the specimen is moved vertically from the parallel beam at the projection angle θ, while the parallel translation means that the specimen is moved toward to or away from the parallel beam. These two types of errors let us distinguish whether a certain error can be corrected or not, because the error occurring horizontally to the beam is hard to notice, and therefore hard to calculate the distance of shift. And significantly, the pattern of sinogram flows continuously showing no discontinuity in parallel translation (Fig. S2a).

2.2. An optimization algorithm of translation error

The error occurring vertically to the beam shows a definite cut-off point as the graph of Fig. S2b illustrates. In Fig. S2b, the error appeared when the parallel beam was shoot perpendicularly to the stage and the cut-off of the sinogram was reflected at the 90 degrees of θ. The error that arises when the specimen is moved perpendicularly to the beam is always reflected identically on the projection and the discontinuity of equal amount is also found in the sinogram. As a result, we can spot the exact point where the discontinuity happens and correct this error mathematically.

If we have an orthogonal translation error in the e-th column, the correction process has to start from the point where the error occurred and the rest of sinogram has to be modified accordingly. Let us calculate how far the shadow has to be compensated in order to make this inaccurate sinogram an errorless, ideal one. V_n is the distance to which the n-th column has to be moved in the sinogram, and it is written as follows:

$$V_n = v * \cos(\theta_n - \theta_e), n \geq e \text{ (or } n < e)$$

θ_n indicates the angle of n-th column and θ_e is the angle at which the orthogonal translation error occurred in the sinogram. v is the distance that the object is vertically moved to, and can be measured through the two columns θ_{e-1} and θ_e in the sinogram, for the sinogram exactly reflects the shift. To find v, we used a certain section of e-th column and the section will be compared to the part of $(e-1)$-th column. Here, calculating v is an issue of the optimization problem and $f_e(t)$has an absolute minimum at $t = v$ and $n = e$.

$$f_n(t) = \frac{1}{m}\left\{\sum_{k=1}^{m} |p_{n-1}(k+s-1) - p_n(k+s-1+t)|\right\}, -s < t < s$$

A total pixel number m is used in calculation. s is the starting point in the $(e-1)$-th column. $p_i(j)$ indicates the value of a pixel position at i-th column and j-th row in sinogram; $p_1(1)$ indicates the initial pixel point. If the object's shadow length in the sinogram is small enough compared to the projection size, $m = \frac{1}{2} * ny$, $s = \frac{ny}{4}$ is enough to use (ny is the total vertical pixel number). t is an integer and indicates translation. To simplify the calculation, we assumed that the pixel length is the unit length. To obtain the real value of v, it is possible to use the symmetric axis of a quadratic equation with $v-1$, v, $v+1$ and their f_n.

For example, the values of $v = 49$, and $\theta_e = 90°$ were obtained when the sinogram of Fig. S2b was analyzed. Any abnormal peaks were checked to figure out $\theta_e$in the graph where x-axis indicates the angle and y-axis indicates the average difference value v, which is the distance

between two columns of $t = 0$. After finding θ_e, we calculated v by the minimum value of t, $0 \leq t \leq 60$. Using the vertical movement value driven from the following formula, $V_n = 49 * \cos(\theta_n - 90°)$ $(\theta_n \geq 90°)$, the sinogram of Fig. S2b was transformed to an ideal one shown in Fig. S1a. Generally, it will be transformed to an similar-to-ideal one which satisfies the optimization formula, because we don't exactly know the column information of the ideal sinogram at θ_e. Fig. S4a,b shows how we can apply the orthogonal translation algorithm to the real projections.

If only we know the angle θ at the moment of parallel translation error, we can figure out the distance of error using a similar technique we used for vertical translation error compensation; the parallel translation error is corrected in the same way. The formula for this is presented below. (See the Fig. S2a)

$$P_n = p * \sin(\theta_n - \theta_e)\,, n \geq e \text{ (or } n < e)$$

P_n is the distance to which the n-th column has to be moved in the sinogram.

2.3 The limit of Optimization

In our study, we realized that every single point on the stage shows a periodic motion around the RA and the motion will be reflected as a sinusoidal function on the sinogram. If we try to optimize the errors, the optimization was only done to the moment of the error occurred in the sinogram, and the sinogram was vertically moved to connect the discontinuity without talking the sinusoidal function into account. This, unfortunately, leads to flawed image reconstruction. So, we had to use a formula like V_n to modify our sinogram. This is shown quite intuitively in Fig. S3. An object was placed on the exact center-of-rotation, and the ideal sinogram of the object is found in the Fig. S3a. If there is a vertical error at the 90 degrees, the sinogram will be like Fig. S3b, and the error will be also seen in the reconstructed image. Simply moving vertically and linking the sinogram will bring us a sinogram like Fig. S3c and it will still yield incorrect, but different kind of reconstructed image. Our formula V_n, when the sinogram after the error is corrected, will bring a correct reconstructed image that is the same with the original image. Yet, it is not considered as a perfect reconstruction because what we used is the information of θ_{e-1}, not the exact point of error, θ_e, in optimization with the formula V_n. Therefore, we eventually need the function $\boldsymbol{T}_{r,\varphi,\mathrm{h}}$, using CA or FP.

3. Tilting error

3.1 The need to distinguish the tilt of RA and the one of the object in tilting errors.

If a cylindrical object on the stage is tilted and projected, we cannot tell whether it is the object or the RA of the stage that is tilted, only by looking at the projection image. However, those two should be distinguished and defined. When the RA is upright and only the object is tilted, it should not be called a tilting error and consequently, the projection set should not be corrected. Instead, it should be considered as we put some other object which originally has a tilted shape. As it sounds ironical, one would hardly get an ideal projection set if this kind of error is corrected because the original projection set was already correct. Nevertheless, it is hard to tell the difference between the tilt of an object and the one of a RA in the projection image; one should turn to the sinogram in this case because these errors will be seen more easily and clearly in the sinogram.

3.2 Categorization of tilting errors

It is the projection image of a cylinder standing perpendicularly on the RA of the stage in the Fig. S4a. Fig. S4b shows a projection image of the cylinder that is leaned in parallel with the beam and its depth of the shadow is changed, meaning the information of the image is changed. One can see the projection image of the same cylinder tilted vertically in Fig. S4c. It is shown that the depth of shadow remains unchanged, even though the object is tilted; the projection information is not changed.

4. Rotation errors

When we have a rotation error, some information of the image projected from certain angles is lost when the angles of x-ray beam and the rotation overlap. For instance, only the same face of an object is projected even though the beam supposedly takes every different projection, when the rotation of specimen itself countervails that of the beam. In this case, we are not able to obtain every projection image of every angle, namely a complete projection set, which is an essential element in x-ray tomography. And therefore, we will not discuss further on the issue in this study.

5. The linearity of the relationship between x-ray absorption and the thickness of an object.

As other studies have pointed out, most of the objects maintain the linear relationship in the soft x-ray area. We should be able to modify them if it is the object that does not present linear relationship or it is in other x-ray area than soft x-ray. If the material that consists of the specimen is relatively identical and the x-ray attenuation function against length is a one-to-one function having the single attenuation coefficient (AC), the linearity can be granted with a simple modification. If the AC is composed of materials that are not identical, what we can do is to ensure the linearity to the projected image of an object that has the shortest section of linearity guaranteed.

The CA in our study is expected to significantly contribute to a better image reconstruction in x-ray tomography and to be utilized as a versatile tool. Nevertheless, there is a definite limit. For instance, when the specimen is mixed up with materials having greatly diverse ACs to the extent where it is out of linearity, it is hard to compensate the nonlinear area and this may result in errors. And it is not beneficial to use the CA in those cases when the specimen is projected with impermeable materials like metal to make it distinguished. It is better to project the specimen itself without any other distinguishing material when anyone wants to use CA in image reconstruction. The ring artifact due to the CCD defect could be another hurdle in CA application. We need to correct the ring artifact beforehand; the correction itself can also raise some changes in reconstruction errors.

To obtain well reconstructed image, we should make sure to get a consistent x-ray density against the object regardless of projecting angles. In reality though, we sometimes get the different x-ray density according to the projecting angle changes, and then, the value of A also should be changed in the formula applying the CA. It would be difficult to generally use CA for the reconstruction if the location of CA depends on the changes of x-ray density and the A value. The changes of x-ray density (according to the angle) will appear in the sinogram in forms of the shadow intensity. Nonetheless, it won't affect the pattern of the sinogram. In Fig. 2, the pattern of ideal sinogram was maintained although there was a 50% decrease in x-ray density from the 90 degrees of θ. The reconstructed image using the sinogram with the CA and the $\mathrm{T}_{\mathrm{r},\varphi,\mathrm{h}}$ function applied (Fig. 2e) showed no difference in terms of image itself when compared with the reconstructed image of Fig. 2d, and the image of specimen in Fig. 2e was laid in the center. This proves to us that the location of CA does not vary despite the changes of A value against each θ drawn by the different x-ray density. Also, we may be able to get a better image if we mathematically modify the x-ray density in order to even up the A value.

Supplementary Figures (S1-S7)

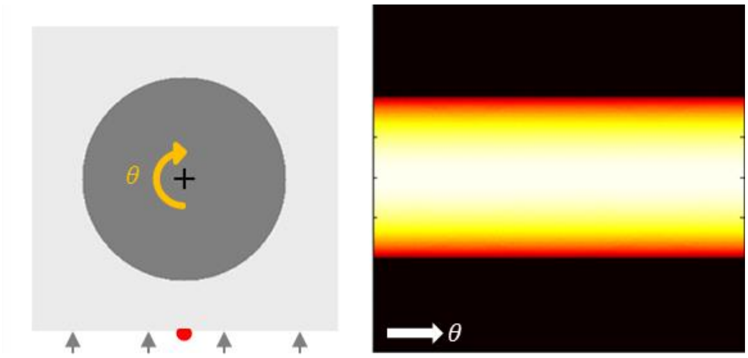

a

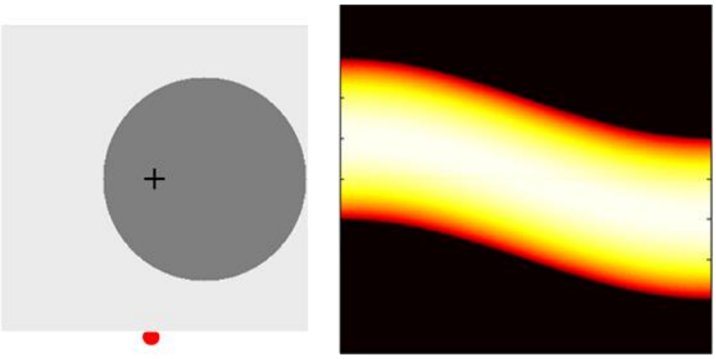

b

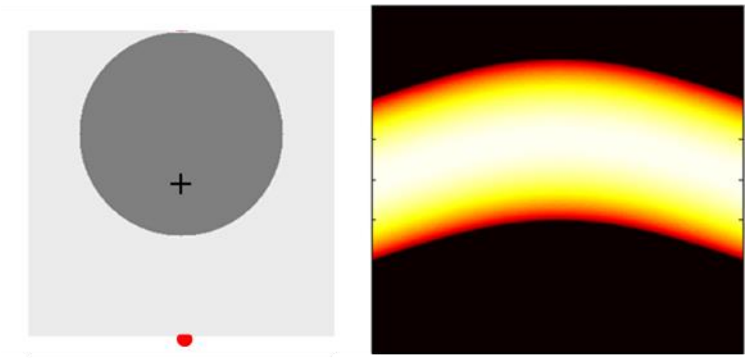

c

Figure S1| The sinograms of specimen that were placed on several different part of the stage. Notice that we marked the stage with the red dot at the bottom to indicate θ is zero degree. **a**, The sinogram when the specimen rotates on the center of the stage. **b**, The sinogram when the specimen is translated in parallel with the beam at $\theta = 0°$. **c**, The sinogram when the specimen is translated vertically to the beam at $\theta = 0°$

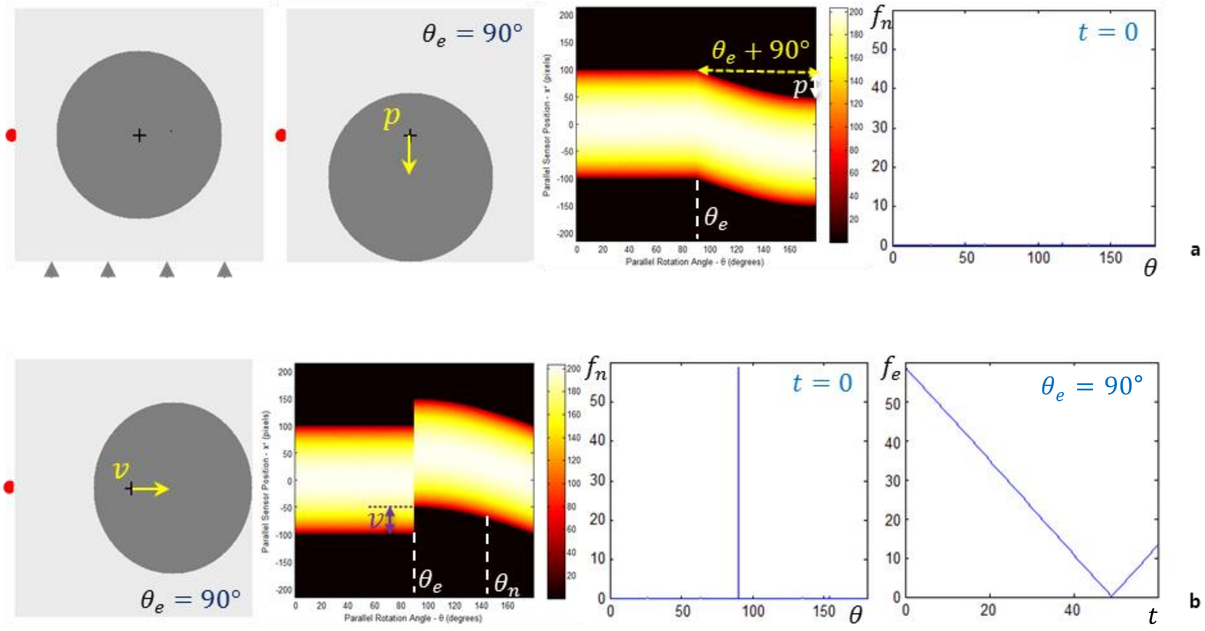

Figure S2| The sinograms with the translation errors during the beam time. a, When the specimen is translated in parallel with the beam at θ=90°, the pattern of sinogram shows a change, but flows continuously. The graph which illustrates the value change of $f_n(t)$ to the angle, θ, doesn't have any discontinuity, therefore, it is hard to spot the specimen. **b**, When the specimen is translated vertically to the beam at θ=90°, there is a discontinuity in the sinogram pattern. It is the same in case of the graph with the value change of $f_n(t)$ to the angle, θ. The discontinuity is found at $\theta_e = 90°$, and the minimum is found at $t = 49$ in the graph.

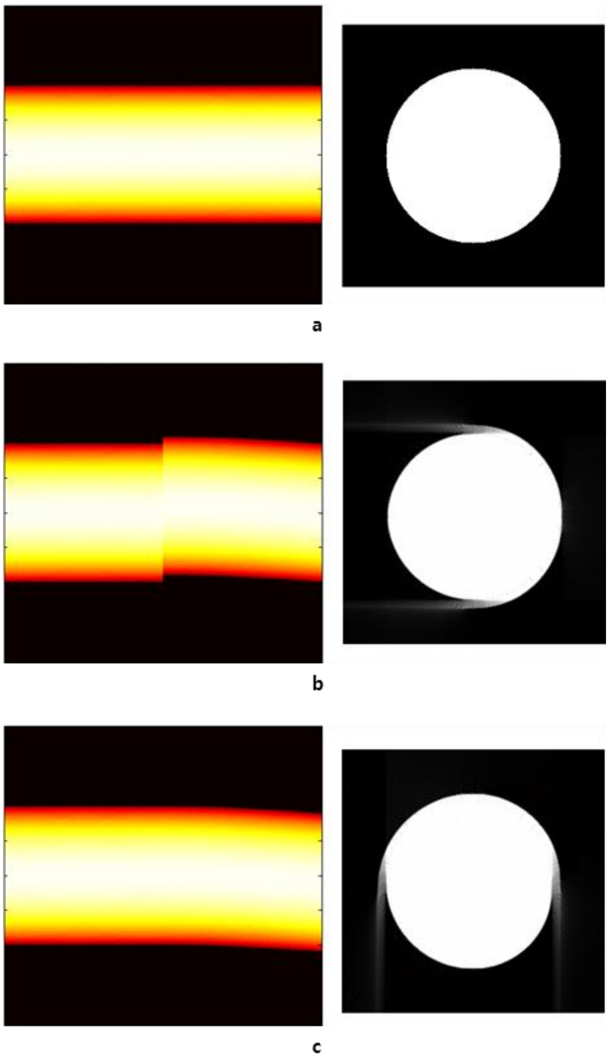

Figure S3| Vertical Translation with Optimization method. a, The ideal sinogram and its reconstruction image that were from the specimen of Fig. S1a. **b**, The sinogram with vertical translation error at $\theta = 90°$ and its reconstruction image. The translation error is found also in the reconstruction image. **c**, The translation error in **b** was optimized meaning that the part of sinogram after the discontinuity was cut and pasted by the distance of translation error in this sinogram. Another error was found in its reconstruction image.

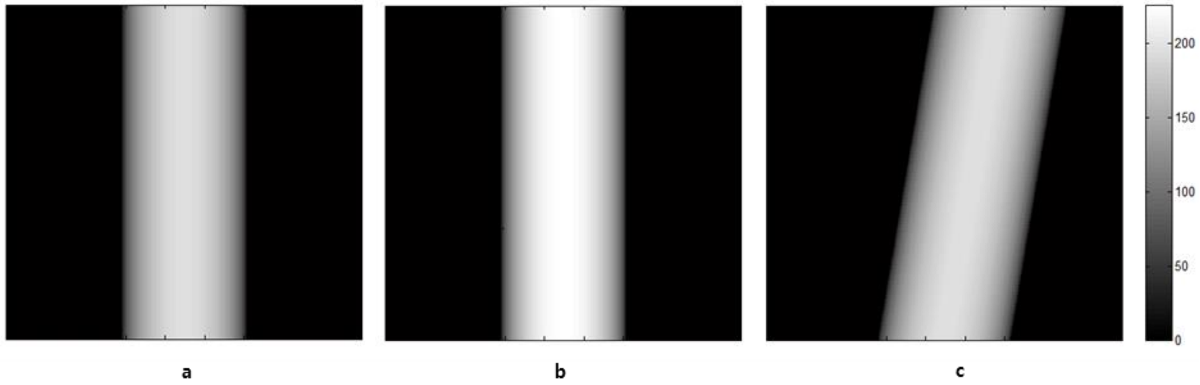

Figure S4| The shadow changes depending on the location of a cylindrical specimen. a, The projection image when the specimen stands upright. When translated without tilts, the shadows are of same shapes even when the locations on the projection are different. **b**, The projection image when the specimen is tilted in parallel with the beam. The shadow is darker than before; the information of the image is changed and the restoration with the projection image is not easy in this case. **c**, The projection image with the vertical tilt of the specimen. The shadow is leaned, however, the information of the image remains the same. In this case, it is possible to restore with the projection image.

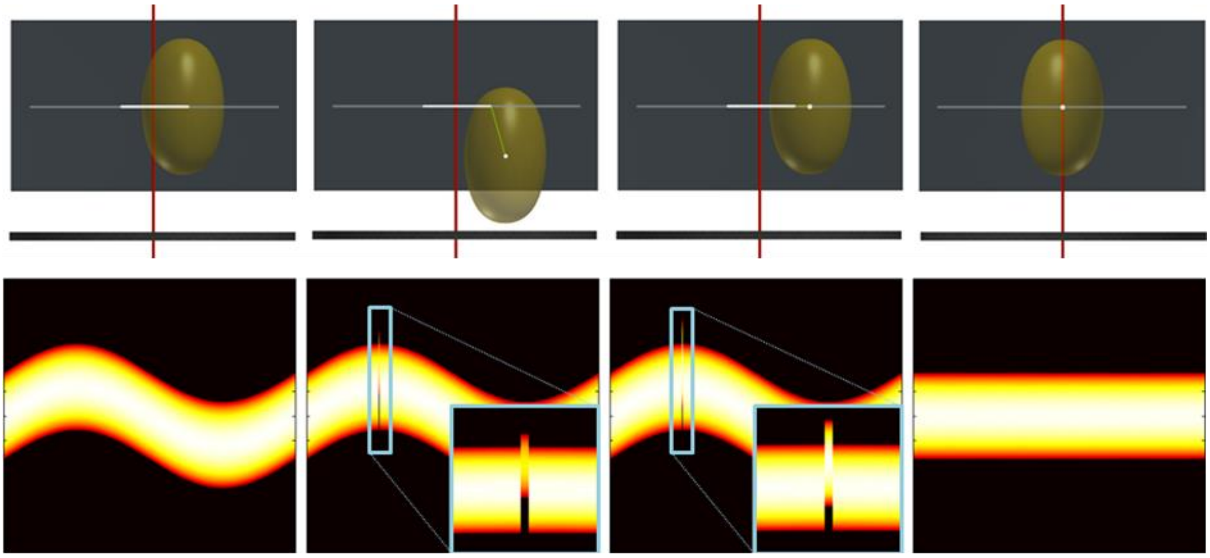

Figure S5| An object of prolate spheroid is located on the rotating stage, which has the center of CCD as the RA, and rotates for the 360 degrees. The CA is at the height of $\boldsymbol{h = 0}$, and the distance from the center is $\boldsymbol{r = d}$. The azimuthal angle of the object is 0 degree when the $\boldsymbol{\theta}$ is zero. The white dot and the line represent where $\boldsymbol{\overrightarrow{P_{CA}}}$ lies against each $\boldsymbol{\theta}$. a, It is the sinogram of the $h = 0$ layer when the object is at $\theta = 90°$ and there is not any known error. **b**, It is the sinogram of the $h = 0$ layer when there was a translation error at $\theta = 90°$. The location of the object in the involved layer is moved to the lower left on the stage and its shape is narrower, compared to that of **a**. This is reflected in the sinogram; the location of shadow and its width have changed (Look at the magnified part of the sinogram). **c**, The $\overrightarrow{P_{CA}}$ in the projection of b was translated vertically to the stage, exactly on the layer in which the $\overrightarrow{P_{CA}}$ without error exists, $h = 0$. The location of shadow has not changed, however, the width now becomes identical with the other θs in the sinogram. **d**, When we aligned $\overrightarrow{P_{CA}}$ on the function $\mathrm{T}_{0,\varphi,0}$, the $\overrightarrow{P_{CA}}$s were all gathered onto one dot on the center of the stage, and the sinogram became linear.

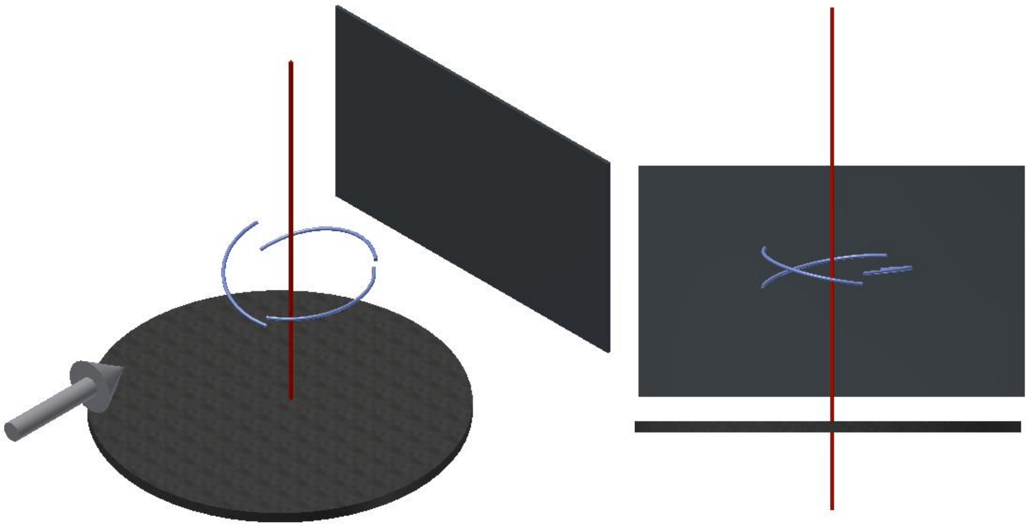

Figure S6| The trajectory of fixed point when there were several errors during the beam time. When the translation error and tilting error occurred partially, we can get the RA of each piecewise continuous section by analyzing the trajectory of fixed point in each section. We can obtain the well reconstructed image when we modify the projections using these RAs.

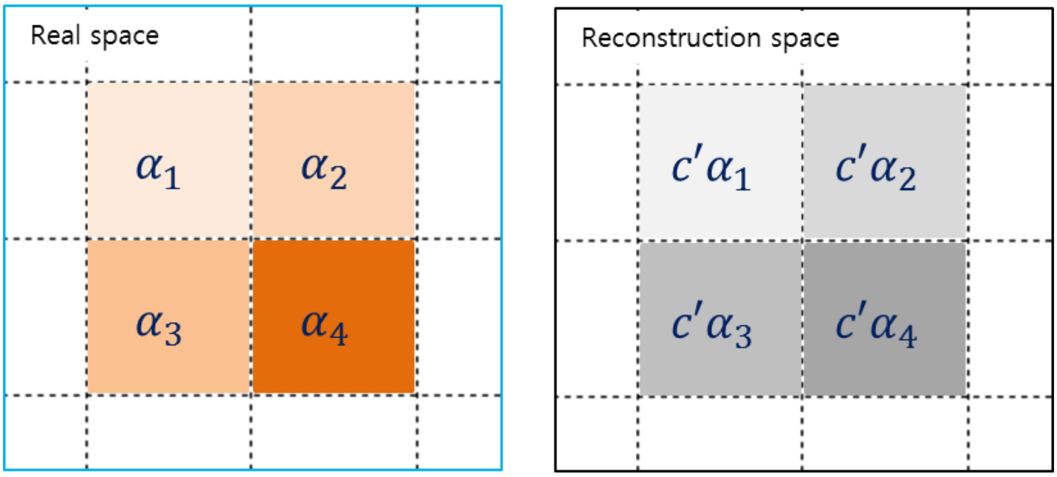

Figure S7| Our hypothesis for the reconstruction between the real specimen and the ideal reconstruction image.
